# The association of healthy lifestyle behaviors with mental health indicators among adolescents of different family affluence in Belgium

**DOI:** 10.1186/s12889-020-09102-9

**Published:** 2020-06-18

**Authors:** L. Maenhout, C. Peuters, G. Cardon, S. Compernolle, G. Crombez, A. DeSmet

**Affiliations:** 1 Department of Movement and Sports Sciences, Faculty of Medicine and Health Sciences, Ghent University, Ghent, Belgium; 2 Department of Experimental-Clinical and Health Psychology, Faculty of Psychology and Educational Sciences, Ghent University, Ghent, Belgium; 3 Faculty of Psychology and Educational Sciences, Université Libre de Bruxelles, Brussels, Belgium; 4 Department of Communication Studies, Faculty of Social Sciences, University of Antwerp, Antwerp, Belgium

**Keywords:** Mental health, Adolescent, Family affluence, Healthy lifestyles, Physical activity, Breakfast, Smoking, Alcohol, Sleep

## Abstract

**Background:**

Healthy lifestyles may contribute to better mental health, which is particularly important in adolescence, an age at which half of all mental health problems first occur. This association may be even more relevant in adolescents of low family affluence, who show more mental health problems, as well as more unhealthy lifestyles. This study investigated healthy lifestyle behaviors, namely sufficient sleep and physical activity, daily breakfast intake, low levels of alcohol use or smoking, in relation to mental health and symptoms of mental health problems (feelings of depression, anxiety, stress and self-esteem) among adolescents from different family affluence. Furthermore, the moderating role of family affluence was examined in those relations.

**Methods:**

Adolescents aged 12-18y were recruited via a random sample of schools in Flanders, Belgium. A total of 1037 adolescents participated (mean age = 15.2, 49.8% female). Independent samples t-tests, Mann Whitney U-tests and χ^2^-tests determined the differences in healthy lifestyle behaviors and mental health indicators between adolescents of low-medium and high family affluence. Regression analyses assessed the association between healthy lifestyles and mental health outcomes and the moderating role of family affluence.

**Results:**

All healthy lifestyle behaviors were associated with at least one mental health outcome, with the exception of alcohol consumption. Adolescents from low-medium family affluence had lower levels of physical activity, less often took breakfast, had lower levels of alcohol consumption and reported lower self-esteem than adolescents from high family affluence. The results showed no moderating effect of family affluence for the association between healthy lifestyle and mental health.

**Conclusion:**

These findings support the value of integrating healthy lifestyle behaviors in interventions for mental health promotion, for both youth of low-medium and high family affluence.

## Background

Youth mental health is defined by the World Health Organization as a state of well-being allowing youngsters to learn and acquire education, have a positive sense of identity, manage their thoughts and emotions, have a fulfilling social life and full participation in society [1]. Mental health thus exceeds the absence of mental disorders or disabilities [1]. Instead, it is a dynamic ability to find a balance between all aspects of life [2]. Poor mental health is a severe public health concern, particularly in adolescents. Half of all mental health problems start by the age of 14, making adolescence a crucial period for mental health promotion [3]. Worldwide, it is estimated that 10**–**20% of adolescents experience mental health problems [4]. In Belgium, 80.9% of adolescents between 2 and 18 years of age do not report an emotional problem (defined as a mental health problem with clear, excessive and persistent signs of depression, anxiety, panic, phobia or other emotional problems), 9% is considered a borderline case and 10% has a suspected pathology [5]. Evidence-based treatments for mental health problems, such as psychotherapy and pharmacotherapy, form a cornerstone of care available for those suffering from mental health problems. Such treatments, however, also face challenges: only small effects of treatment were found for a large group of patients [6, 7], young people are often reluctant to seek professional help [8], treatment comes at a high price for individuals and health care systems, and such treatments may have a range of undesirable side-effects [9]. In this context, prevention of mental health problems is of utmost importance.

Mental health is influenced by many factors, including everyday behaviors that can be altered by individuals [10, 11]. Mental health programs that support adolescents in managing their mental health by improving everyday health behaviors, are empowering, destigmatizing, and can have a large impact at population level at low cost [6]. Several modifiable risk and protective factors have been identified in adolescents: sufficient sleep and physical activity and a healthy diet were associated with better mental health outcomes, such as lower depression, anxiety, stress [6, 11–16] and higher self-esteem [14, 15, 17–20]; whereas high alcohol consumption and smoking were associated with less beneficial mental health outcomes, such as higher psychological distress, depression, anxiety, stress [6, 21–23], and lower self-esteem [24, 25]. There is room for improvement in the area of healthy lifestyles among Flemish adolescents: half do not meet the norm of 8-h of sleep; most do not engage in sufficient physical activity; and around half do not take breakfast daily [26–30]. Although the rate has slightly decreased, a substantial number of adolescents engages in drinking (34.4–36%) and smoking (2.4–4.3%) [31, 32]. Increasing healthy behaviors (sufficient sleep and physical activity, and a healthy diet) and reducing these unhealthy behaviors (alcohol consumption and smoking) can increase adolescents’ resilience and mental well-being.

In most countries, adolescent health outcomes are associated with socioeconomic status (SES) [33–37]. Adolescents of low SES thus form a particular group of interest for promoting mental health via healthy lifestyles. Both mental health and healthy lifestyles in adolescents of low SES are found to be poorer than among adolescents of high SES. A systematic review [38], which included studies that measured SES in various ways (i.e., through parental occupation, income or education), showed that socioeconomically disadvantaged children and adolescents are two to three times more likely to develop mental health problems [38–40] than their peers from socioeconomically advantaged families. In addition, adolescents from low SES reported lower levels of sleep, physical activity and healthy diet, and higher levels of smoking than teens from high SES [28–30, 32, 34, 41–50]. Research on how adolescents’ alcohol consumption differed as a function of SES was inconsistent [31, 47, 49, 51–53]. It can be expected that unhealthy lifestyles have an even stronger association with mental health in adolescents of lower SES than of higher SES, since both healthy lifestyles and mental health are found to be poorer in this population. To our knowledge, only two studies so far have addressed the associations between healthy lifestyle behaviors and mental health in adolescents of low SES. These studies showed positive associations, but focused on only one health behavior (i.e., physical activity or sleep) in relation to psychological distress [43, 54]. The present study combines different health behaviors and includes positive well-being in defining mental health. Moreover, SES is measured using the Family Affluence Scale [55], as this can be easily answered by adolescents themselves. Different ways exist to measure ‘SES’. Many researchers refer to the general term ‘SES’, but use specific indicators (i.e. income, occupation, education, etc.). This makes it difficult to compare study results. When referring to our specific SES-indicator (i.e. FAS), we have therefore consistently used ‘FAS’ to be specific and clear. However, when we are referring to other studies or comparing our results with previous studies, we will use the general term ‘SES’, since other researchers have used different indicators to refer to SES. The aim of the current study is to investigate: 1) associations between healthy lifestyles, namely sufficient sleep duration and physical activity, daily breakfast intake, low levels of alcohol use or smoking, and mental health (research question RQ1); 2) the level of healthy lifestyle behaviors and mental health among adolescents from different family affluence (RQ2); and 3) the moderating role of family affluence in the relation between healthy lifestyles and mental health (RQ3). It is hypothesized that healthier levels of lifestyle behaviors are associated with better mental health (H1); that adolescents from lower family affluence report lower levels of healthy lifestyles and poorer mental health than adolescents of higher family affluence (H2); and we expect to find that family affluence plays a moderating role in the relation between the investigated healthy lifestyle behaviors and mental health outcomes (H3). Our results may indicate which lifestyle behaviors are associated with adolescent mental health, and may encourage health professionals in designing programs to lower the risk for mental health problems among adolescents. In addition, these results can shed a light on health promotion strategies particularly effective in lower family affluent groups in youth.

## Methods

### Participants and data-collection

We selected a random sample of schools (*n* = 26) from a government database of secondary schools in Flanders, a region in Belgium consisting of around 6 million inhabitants. All included schools were state-funded, as the vast majority of schools in Belgium are state funded. Eight schools (31%) agreed to collaborate in the study. The main reason for not participating was no time to set-up the survey at the school within the time frame of the study. The study took place between November 2014 and May 2015. Within each school, classes were randomly selected. We aimed to collect data among grades 7 to 12 for each school (aged 12–18). Data collection took place at school, during one class hour. The anonymous paper-and-pencil survey was administered by the researchers, who explained at the start of the survey that students were under no obligation to participate and could withdraw at any time. Students were assured that their responses would be confidential and that no information would be shared with teachers, parents, or fellow students. Five students declined to participate, none of the parents declined consent. The study received approval from the Ethics Committee of the Ghent University Hospital (2012/307, B670201214183). Adolescents provided written informed consent, parents provided passive informed consent. Parents were informed about the study through the school and received a telephone number and e-mail address from the researchers, via which they could notify that they did not want their child to participate. They were informed that when they did not contact the researchers, they agreed with participation of their child.

### Measures

#### General socio-demographic information

Items were selected from the Health Behavior in School-aged Children (HBSC) 2009/10 questionnaire, a cross-national survey supported by the World Health Organization [56]. Socio-demographic variables included gender, age, type of education (general academic, technical or vocational track), country of birth, family living situation, self-reported weight and height (used to calculate Body Mass Index, BMI). Age, gender and BMI were taken into account as covariates in this study, as gender, age and BMI differences have been associated with adolescents’ health behaviors and (mental) health outcomes [57]. For example, girls are less likely to have breakfast every weekday and boys, in general, report early and weekly smoking more often. In addition, girls are more likely to describe lower life satisfaction in comparison to boys [57]. Furthermore, a negative drop in healthy behaviors is seen with increasing age. For example, 11-year-olds are more likely to meet the physical activity guidelines of at least 60 min of moderate to vigorous physical activity daily than 15-year-olds in almost all countries and regions [57]. BMI has also been reported as inversely associated with global self-esteem in adolescents [58].

##### Family affluence

This part of the HBSC questionnaire also consists of the validated adolescent self-report ‘Family Affluence Scale’ (FAS), to identify family material wealth and socio-economic status (SES) of children and adolescents [55, 59–61]. The FAS is used as an indicator of SES. It has widely been used to explore and explain socioeconomic inequalities in a wide range of health indicators in the HBSC study over the last 20 years [60]. FAS is validated against other measures of SES and macro-economic indicators (e.g. Gross Domestic Product (GDP)) in 35 countries [55, 60]. The FAS was developed to overcome the problem of inaccurate perceptions and missing data among children and adolescents of their family’s finances, especially among lower socio-economic groups which could thus lead to an underestimation of socioeconomic inequalities [36, 60]. It was proposed as a less intrusive, more comprehensible approach to identify the family’s socioeconomic status [62] than inquiring about parents’ educational, occupation or income levels [55, 63]. It is indicated that in contrast to for example parental occupation, the proportion of missing data on FAS items is low [60]. The FAS II consists of four items: number of cars, own bedroom, computers owned and number of holidays per year [55, 62]. A composite FAS score (ranging from 0 to 9) is calculated for each adolescent based on his or her responses to these four items. The following, international, cut-off points were used: score of 0, 1, 2 classified as low affluence; score of 3, 4, 5 as medium affluence; and a score of 6, 7, 8, 9 classified as high affluence [55].

### Healthy lifestyles

Items to assess healthy lifestyles, except for sleep duration, were also taken from the HBSC survey. Several health-related lifestyle behaviors among adolescents are interrelated. Based on Principal Component Analyses on these data reported elsewhere [12], healthy lifestyles were grouped into two factors: ‘energy-balance related behaviors’, consisting of physical activity and a healthy diet, and ‘addictive behaviors and sleep duration’, consisting of alcohol consumption, smoking and perceived sleep duration. These factors will be used to discuss the results, individual behaviors are however retained in the analyses.

### Energy-balance related behaviors

*Physical activity* was measured by the number of days they achieved ≥60 min of moderate to vigorous physical activity, and was defined in the questionnaire as: “bodily movements that make your heart beat faster and make you feel out of breath at some moments”. A *healthy diet* was measured by assessing the number of days per week adolescents had have breakfast. Eating a regular, healthy breakfast contributes to the daily recommended intake of essential nutrients [64, 65]. Moreover, a daily breakfast may be used to identify adolescents at risk for unhealthy lifestyle behaviors. For example, daily breakfast intake is associated with both daily fruit and vegetable consumption, and there is an inverse relationship between daily breakfast intake and daily soft drink consumption [50].

### Addictive behaviors and perceived sleep duration

*Alcohol use* was assessed by summing the frequency of six different types of alcohol consumption: beer, wine, spirits/liquor, alcopops and any other drink that contains alcohol (0-never; 4-daily. Range of summed score 0–24). Three questions were asked on *tobacco use*, based on the Flemish version of the HBSC 2009/10 questionnaire: 1) have you ever smoked tobacco?; 2) how often do you smoke currently?; 3) how many cigarettes per day have you smoked on average over the last 30 days?. These questions were combined and recoded to form one indicator of tobacco use frequency, namely: 0 ‘never smoked’; 1 ‘I have smoked but do not smoke now’; 2 ‘I smoke now, but I am not a daily smoker’; 3 ‘I smoke daily, but I am low dose smoker’; and 4 ‘I smoke daily and am a high dose smoker’. To decide on low dose and high dose among daily smokers, the median among the group of daily smokers was used as a cut-off for tobacco use frequency. The median showed around half of daily smokers smoked fewer than 11 cigarettes per day (=low dose), and half smoked 11 cigarettes or more per day (=high dose). Self-reported smoking is a reliable indicator of smoking status [66]. All questions from smoking were derived from the HBSC study where items have been decided by an international expert team and have been used in numerous studies [67–69]. Specifically, for the three questions on smoking ICC-values of 0.75, 0.50 and 0.85 have been reported [70]. To calculate *sleep duration* (number of hours slept per night), adolescents were asked to report at what time they usually go to bed and get up. Self-reported duration of sleep is strongly correlated with sleep duration measured by accelerometers for weeknights and moderately correlated with sleep duration for weekend nights [71].

### Mental health

Mental health was measured through feelings of depression, anxiety and stress and self-esteem. *Feelings of depression, anxiety and stress* were measured with the Depression Anxiety Stress Scales (DASS-21) which has good psychometric properties to measure adolescent mental health outcomes [72, 73]. It consists of seven items per subscale [73]. Total scores per subscale were used as dependent variables, with high reliability for each of the subscales (α_depression_ = 0.90; α_anxiety_ = 0.84; α_stress_ = 0.87). Focusing on *self-esteem* is considered a core element of mental health promotion and a fruitful basis for a broad-spectrum approach [74]. Positive global self-esteem was measured by a single item from the Rosenberg Self-Esteem Scale (RES), namely ‘I take a positive attitude toward myself’. Global self-esteem can be measured by a single item [75] and this specific item is a main contributor to global positive self-esteem [76, 77].

### Analysis

Variables were checked on normal distribution with the values for skewness and kurtosis. The values for skewness and kurtosis between − 2 and + 2 are considered acceptable in order to prove normal univariate distribution [78]. To test the significance of difference of the health behaviors and mental health indicators between low-medium and high FAS (Table 1), independent samples t-tests were used for variables with a normal distribution (i.e., physical activity, healthy diet, sleep duration, alcohol consumption and self-esteem) and the non-parametric variant, Mann-Whitney U tests were used for the variables that did not have a normal distribution (i.e., symptoms of depression, anxiety and stress). For smoking, a χ^2^-test was conducted. Gamma regression models were used to account for the positively skewed distribution of the dependent variables: depression, anxiety and stress [79]. The dependent variable ‘self-esteem’ showed a normal distribution. For this variable, multiple linear regression analysis was used. Regression analyses assessed the association between healthy lifestyles and mental health outcomes (RQ1); and the moderating role of family affluence in the relation between healthy lifestyles and mental health outcomes (RQ3). Analyses were controlled for individual background factors that significantly influenced mental health outcomes (namely, gender, age and BMI). Analyses were conducted stepwise, by first examining the influence of family affluence and background variables, next the healthy lifestyle variables, and finally the interaction effects between healthy lifestyle variables and family affluence. Because no interaction effects were significant, the parsimonious model was constructed based on the full model of the direct effects (see Tables 2 and 3). In the first regression analyses (Table 2), BMI was not a significant predictor for any mental health outcome and therefore not included in further regression analyses (Table 3). Collinearity diagnostics were conducted examining Variance Inflation Factor (VIF) (≤10) and tolerance (≥0.1). VIF showed no multicollinearity among independent variables. Cross-tabulations were checked for empty combinations of cells or low expected frequencies [80]. Continuous independent variables were mean centered. Moderator variables were created by multiplication of interaction variables. All analyses were conducted in SPSS 25.0.

**Table 1:** Sample characteristics and differences between (in) dependent variables

	**Full sample (*n* = 1037)**	**Low-med family affluence (*n* = 179)**	**High family affluence (*n* = 838)**	**Significance of difference**
Physical activity				
≥ 60 min. Physical activity (number of days/week), mean ± SD	3.33 ± 2.09	2.83 ± 2.07	3.40 ± 2.08	t(1, 990) = − 3.32** ^a^
Healthy diet				
Breakfast (number of days/week), mean ± SD	5.38 ± 2.19	4.88 ± 2.38	5.50 ± 2.13	t(1, 999) = − 3.16** ^a^
Alcohol consumption				
Frequency of alcohol consumption (0–24), mean ± SD	3.36 ± 3.85	2.82 ± 3.60	3.45 ± 3.86	t(1, 994) = − 1.96* ^a^
Smoking				
Percentage (%) current smokers (daily + non-daily)	12.4	14.6	11.9	χ^2^ = 0.95 ^c^
Percentage (%) high dose daily smokers	4.4	7.0	4.0	χ^2^ = 3.05° ^c^
Perceived sleep duration				
Average hours of sleep/night, mean ± SD	7.87 ± 1.42	7.95 ± 1.55	7.85 ± 1.39	t(1, 984) = 0.83 ^a^
Mental health outcomes				
Symptoms of depression, mean ± SD	6.18 ± 8.51	6.80 ± 9.35	6.09 ± 8.34	Z = -0.29 ^b^
Anxiety, mean ± SD	5.44 ± 7.12	5.60 ± 7.23	5.45 ± 7.09	Z = − 0.10 ^b^
Stress, mean ± SD	7.93 ± 8.11	8.30 ± 8.23	7.92 ± 8.10	Z = − 0.51 ^b^
Self-esteem, mean ± SD	3.71 ± 1.09	3.52 ± 1.15	3.76 ± 1.06	t(1, 973) = − 2.6** ^a^

° *p* ≤ .1; * *p* ≤ 0.05; ** *p* ≤ .01; *** *p* ≤ .001

^a^independent samples t-tests

^b^Mann Whitney U-tests

^c^χ^2^-tests

**Table 2:** Regression analysis on the moderating role of family affluence in the relation between addictive behaviors and sleep, and mental health outcomes

	**Dependent variable: mental health outcomes**			
	**Depression**^**a**^	**Anxiety**^**a**^	**Stress**^**a**^	**Self-esteem**^**b**^
**Parsimonious model results (direct effects)**	*BIC = 4753.20* *CAIC = 4758.20*	*BIC = 4721.28* *CAIC = 4726.28*	*BIC = 5694.01* *CAIC = 5699.01*	*F(3, 938) = 21.26, p < .001, adj. R*^*2*^ *= 0.06*
	**Exp(B), (95% CI)**			**β (B; SE)**
Gender (ref. girls)	0.68 (0.56; 0.82) ***	0.66 (0.55; 0.79) ***	0.72 (0.61; 0.84)***	0.19 (0.41; 0.07)***
Family Affluence	*Not included*	*Not included*	*Not included*	0.10 (0.29; 0.09)***
Smoking	1.15 (1.05; 1.27)**	1.17 (1.07; 1.28) ***	1.16 (1.07; 1.26)***	*Not included*
Sleep	0.86 (0.80; 0.92) ***	0.87 (0.82; 0.93) ***	0.91 (0.86; 0.97)**	0.15 (0.12; 0.02)***

° *p* ≤ .1; * *p* ≤ 0.05; ** *p* ≤ .01; *** *p* ≤ .001

^a^Gamma generalized linear model

^b^General linear model

**Table 3:** Regression analysis on the moderating role of family affluence in the relation between energy-balance related behaviors and mental health outcomes

	**Dependent variable: mental health outcomes**			
	**Depression**^**a**^	**Anxiety**^**a**^	**Stress**^**a**^	**Self-esteem**^**b**^
**Parsimonious model results (direct effects)**	*BIC = 4619.84* *CAIC = 4625.84*	*BIC = 4605.30* *CAIC = 4611.30*	*BIC = 5574.12* *CAIC = 5579.12*	*F (3, 951) = 20.12, p < .001, adj. R*^*2*^ *= 0.06*
	**Exp(B), (95% CI)**			**β (B; SE)**
Age	*1.05 (1.00; 1.11)*°	*1.04 (0.99; 1.10)*	*1.04 (1.00; 1.09)°*	*Not included*
Gender (ref. girls)	0.69 (0.57; 0.85)***	0.68 (0.56; 0.81)***	0.72 (0.61; 0.85)***	0.18 (0.39; 0.07)***
Family Affluence	*Not included*	*Not included*	*Not included*	0.08 (0.22; 0.09)*
Physical activity	0.92 (0.88; 0.97)***	*0.96 (0.92; 1.01)*°	*Not included*	*Not included*
Days of breakfast	0.95 (0.91; 0.99)*	0.93 (0.89; 0.97)***	0.94 (0.91; 0.98)**	0.15 (0.07; 0.02)***

° *p* ≤ .1; * *p* ≤ 0.05; ** *p* ≤ .01; *** *p* ≤ .001

^a^Gamma generalized linear model

^b^General linear model

## Results

The initial sample consisted of 1062 adolescents, from which 25 were removed due to incomplete or unsatisfying answers (no variation on relevant diverging questions or nonsense answers to open-ended questions), resulting in an analyzed sample of 1037 adolescents (49.8% female; mean age = 15.17y ± 1.86; mean BMI = 19.56 ± 3.70). For 20 adolescents, no FAS score could be calculated due to missing information. The majority of the adolescents had a high family affluence (82.4%), which is in line with the high affluence rate in this region reported in the HBSC 2009/10 study (i.c. 72.7%; Buijs, T., personal communication). Most adolescents were born in Belgium (94.0%). Around two third lived with both parents (64.1%), one third had another family situation (e.g. living with one parent or in co-parenthood, living with other family members).

Results on sample characteristics can be found in Table 1. On average the sample performed ≥60 min. of moderate to vigorous physical activity on 3,3 days a week; they took breakfast on 5,38 days a week; and slept 7.87 h per night. Moreover, the sample had a relatively low frequency of alcohol consumption (sum score 3.36/24) and 12,4% were current smokers. Adolescents of high family affluence were physically active on significantly more days, took breakfast on more days, reported higher alcohol consumption and had higher self-esteem than adolescents of lower family affluence. There were no significant differences in smoking, sleep duration, or other mental health outcomes between adolescents of low-medium and high family affluence (RQ2).

Table 4 (full table in Appendix) shows the main effects of addictive behaviors and sleep on mental health outcomes, as well as the results of the moderating role of family affluence in the relation between these behaviors and mental health outcomes (RQ1 and 3). A lower sleep duration was significantly associated with lower mental health on all studied indicators. More smoking had a main effect on more symptoms of depression, anxiety and stress. Alcohol consumption was not significantly associated with any of the mental health outcomes. No moderating effect of family affluence was found in these relations with mental health outcomes. Family affluence was found as a significant main predictor of self-esteem.

Table 5 (full table in Appendix) shows the associations between the energy-balance related behaviors and mental health, as well as the results of the moderating role of family affluence in the relation between energy-balance related behaviors and mental health outcomes (RQ1 and 3). Daily breakfast intake was associated with higher mental health on all outcomes. Higher levels of physical activity only showed a significant main effect on one of the mental health outcomes, i.c. lower feelings of depression. There was no moderating role of family affluence in the relation between energy-balance related behaviors with any of the mental health outcomes. Family affluence, however, showed a significant association with self-esteem: youth of low-medium family affluence had lower self-esteem than youth of high family affluence.

## Discussion

This study investigated healthy lifestyle behaviors and mental health among adolescents, thereby differentiating between adolescents from low to medium and high family affluence, and examining whether family affluence plays a moderating role in the relation between certain healthy lifestyles and mental health outcomes. Findings indicated that healthy lifestyle behaviors were indeed associated with better mental health outcomes, and that certain but not all healthy lifestyle behaviors and mental health outcomes were lower among adolescents of low to medium family affluence than those of high family affluence. We, however, did not find that family affluence moderated the association between healthy lifestyles and mental health outcomes. This indicated that healthy lifestyles are important in mental health among adolescents for both adolescents of low-medium and high family affluence.

The results show that all healthy lifestyle behaviors were associated with at least one mental health outcome, with the exception of alcohol consumption. Lower sleep duration and daily breakfast intake were significantly associated with lower mental health on all studied indicators. Higher levels of physical activity only showed a significant association with one of the mental health outcomes, i.e. lower feelings of depression. More smoking showed an association with higher levels of feelings of depression, anxiety, and stress, but not with self-esteem. This pattern of results was, however, not completely in line with our idea that all forms of healthy lifestyles would be associated with all of the mental health outcomes (lower feelings of depression, anxiety and stress and higher self-esteem). Only sufficient sleep and daily breakfast intake were related to all mental health outcomes included in this study. It may be that depending on the mental health outcome, other healthy lifestyle behaviors are important. In this sense, combining different healthy lifestyle behaviors in a mental health promotion intervention may be beneficial, as various mental health outcomes are important for a positive mental well-being of adolescents.

The results only partially support the hypothesis that adolescents from lower family affluence would engage in less healthy levels of lifestyle behaviors and experience poorer mental health outcomes than adolescents from higher family affluence. Consistent with previous studies [39, 40, 42, 47], the present study shows that adolescents from low-medium family affluence had lower levels of physical activity, less often took breakfast, had lower levels of alcohol consumption and reported lower self-esteem than adolescents from high family affluence. No significant differences between youngsters of low-medium and high family affluence were found for sleep duration, smoking, and for feelings of depression, anxiety and stress. Regarding sleep and smoking, previous studies reported that adolescents from lower family affluence had poorer sleep duration [41, 43] and higher levels of cigarette smoking [46–49] than adolescents from high family affluence. Our findings regarding sleep duration and cigarette smoking were therefore not consistent with previous studies. In the area of alcohol consumption, previous studies that compared adolescents of low family affluence with adolescents of high family affluence have reported inconsistent results [47, 51–53]. Our findings show a small difference, indicating that adolescents of high family affluence on average reported higher alcohol consumption than adolescents of low-medium family affluence. A possible explanation may be that adolescents with more pocket money are able to buy more alcohol than adolescents with less pocket money [51, 81]. However, other studies also have reported weak, inverse or no links in alcohol consumption between low-medium and high family affluence [47, 52, 53].

Some of the differences between our findings and the literature may be due to different ways of measuring SES. Prior work already indicated that relationships between healthy lifestyles and SES may be inconsistent across SES indicators [34, 38, 49, 53]. In our study, an adolescent self-report measure, namely FAS, was used to identify SES of adolescents [55]. This in comparison with studies that measure (parental) SES through income, education or occupation [41, 43, 48, 49, 51, 52]. FAS measures only one aspect of SES, which is much more related to material wealth, income and spending patterns [34, 82]. In line with our study, for example, Richter et al. reported a small effect of family affluence on alcohol consumption; indicating an increasing risk of higher alcohol consumption with increasing family affluence, whereas no significant association was observed for educational track [53]. Furthermore, physical activity and daily breakfast intake might be influenced more by financial resources (i.e., possibility of registering in a sports club or purchasing healthy food) than sleep or smoking. Those latter behaviors may be more strongly associated with education and occupational status than with income or material wealth [34]. Parental occupation reflects to some degree parents’ educational status. Educational strategies, values, norms and model behavior of parents may be more likely to positively influence sleep or smoking [34, 82]. However, it is difficult to draw a clear line in this, as we can imagine that buying cigarettes in many countries is also expensive, and having a daily breakfast can be strongly influenced by parents’ modelling behavior. Moreover, FAS associations are strong for health outcomes that are related to family culture and behavior (such as physical activity and healthy diet), but less so for some behaviors with strong peer norms (like alcohol use and smoking). Those addictive behaviors (alcohol use and smoking) might be less strongly influenced by parental socioeconomic status [34, 53]. In this study, there was only a small significant difference in alcohol consumption between low-medium and high family affluence. Richter et al. concluded that other determinants (like for example peers or school setting) might have a larger impact on adolescent alcohol consumption than parental SES (measured with the FAS) or adolescents’ own SES (measured through educational track) [53]. In general, it is important that further research explores to what extent the different indicators of SES influence adolescent healthy lifestyle behavior as this could give important insights for preventive strategies [34].

Although this study shows that healthy lifestyles are clearly significant predictors of mental health, and there are some differences in healthy lifestyles according to family affluence, we did not find any significant differences in symptoms of mental health problems (feelings of depression, anxiety and stress) between adolescents from low-medium family affluence and adolescents from high family affluence. Prior work indicates that low family affluence tends to be more strongly related with externalizing problems (e.g., attention deficit hyperactivity disorder, conduct disorder, antisocial behavior) than with internalizing problems (such as depression, anxiety and stress that were included here) among children and adolescents [38]. Moreover, it may well be that other unmeasured factors contribute to mental health. For example, social support from friends and spending time with friends during leisure time are strong protective factors against symptoms of depression and anxiety in adolescents [83], especially in those living in areas of low socioeconomic disadvantage [84]. To conclude, we see that adolescents with low-medium FAS report lower levels of self-esteem, but that this is not the case for symptoms of depression, anxiety and stress. Future research may identify possible underlying reasons.

This study furthermore shows no moderating effect of family affluence. This indicates that the relationships between lifestyle behaviors and mental health outcomes exist for both adolescents of low-medium and high family affluence. This would mean that mental health interventions can focus on the healthy lifestyle behaviors for adolescents of all forms of family affluence. Adolescents of low-medium family affluence may however need more or different support in reaching these healthy lifestyles and mental health outcomes, as they to some extent still report lower levels of healthy lifestyle behaviors and poorer mental health outcomes. Future research needs to explore how such interventions may be best designed.

## Conclusion

Attention should continue to be paid to (mental) health inequalities between adolescents of low-medium family affluence and high family affluence. Poor mental health among adolescents of low-medium family affluence (as of other family affluence) might be reduced by improving health-related behavior. Our study concluded that all included healthy lifestyle behaviors are associated with at least one of the mental health outcomes, with the exception of alcohol consumption. Adolescents can tackle these behaviors in their daily lives to reduce their risk of mental health problems and build their resilience, and should therefore be integrated in interventions for mental health promotion. This was to our knowledge the first study to assess whether family affluence plays a moderating role in the association between these aforementioned different healthy lifestyle behaviors and mental health outcomes. No moderating effect of family affluence was found.

### Limitations and strengths

This study had some limitations. First, our design is cross-sectional in nature. Hence, the causal direction of these relationships cannot be determined. Intervention or longitudinal studies are needed to assess whether healthy lifestyle behaviors have an effect on mental health indicators. Second, there is a wide variety of SES-measures across studies in literature. The inconsistent use of these SES-measures complicates comparisons, explanations and interpretations. Third, the majority of our sample was highly affluent, consistent with the high affluence of the country. This may limit the generalizability of our findings to other countries with a lower national level of affluence. Fourth, the explained variance of the healthy lifestyles in relation to mental health outcomes was quite small. Even though various healthy lifestyle behaviors were analyzed there are, of course, other important (lifestyle) factors that were not included in this study (e.g., social support, (social) media influences, relation with peers, etc.) [83, 85, 86]. Mental health promotion programs may therefore consider to also include other components besides healthy lifestyles. Furthermore, interpreting adolescents’ lifestyle behavior obtained from self-reports can be difficult as these may be influenced by social desirability. Nevertheless, we expect a low social desirability bias given the survey’s anonymity. In line with this, also adolescents’ weight and height for calculating BMI were self-reported; such results may be biased [87]. Despite the widespread use of the four items in FAS II, they may not be bias-free, especially in cross-national contexts. The FAS-items should continue to be updated to reflect material affluence of the family across countries. To reduce the burden for the adolescents, daily breakfast intake was the only indicator of healthy diet. However, a healthy diet consists also of other aspects of nutrition (e.g., fruit, vegetables, fish, etc.) [88–90]. Lastly, our study investigated alcohol consumption as one of the (un) healthy lifestyles. For some adolescents, alcohol consumption may be prohibited by their religion, and this may also impact results. We however did not question adolescents’ religious or cultural restrictions regarding alcohol consumption, this would be a valuable aspect to take into account in future research. The study also had several strengths. First of all, mental health was defined using a broad concept of both positive well-being and mental health problems, in line with the WHO conceptualization of mental health. Family affluence was measured using a validated scale that provides reliable information based on adolescents’ self-reports [55]. The FAS has the advantage that it can be easily answered by youth. Furthermore, the FAS makes international comparisons possible, as this scale is used in all the HBSC-studies across different countries. It is indicated that the FAS may be more ecologically valid than parental income data since it is based on the family context of consumption [55, 63]. Our study added to the scarce research on healthy lifestyles to improve youth mental health and how this differed by youth’s family affluence. Our study showed that healthy lifestyles differ between youth of low-medium and high family affluence and that these healthy lifestyles may contribute to a better mental health for all.

## Data Availability

The datasets used and/or analyzed during the current study are available from the corresponding author on reasonable request.

## Appendix

**Table 4:** Regression analysis on the moderating role of family affluence in the relation between addictive behaviors and sleep, and mental health outcomes

	**Dependent variable: mental health outcomes**			
	**Depression**^**a**^	**Anxiety**^**a**^	**Stress**^**a**^	**Self-esteem**^**b**^
**Full model results (direct effects, only family affluence)**	*BIC = 5007.032* *CAIC = 5010.032*	*BIC = 5007.17* *CAIC = 5010.17*	*BIC = 6010.34* *CAIC = 6013.34*	*F (1, 973) = 7.57, p = 0.006, adj. R*^*2*^ *= 0.01*
	**Exp(B), (95% CI)**			**β (B; SE)**
Family affluence (ref. low/medium)	*0.90 (0.70; 1.15)*	*0.97 (0.77; 1.23)*	*0.95 (0.78; 1.17)*	*0.09 (0.25; 0.09)***
**Full model results (direct effects, all background variables)**	*BIC = 3927.32* *CAIC = 3933.32*	*BIC = 3906.11* *CAIC = 3912.11*	*BIC = 4674.49* *CAIC = 4680.49*	*F(4, 751) = 10.54, p < .001, adj. R*^*2*^ *= 0.05*
	**Exp(B), (95% CI)**			**β (B; SE)**
Age	*1.07 (1.01; 1.15)**	*1.05 (0.99; 1.12)°*	*1.04 (0.99; 1.10)*	*0.00 (0.00; 0.02)*
Gender (ref. girls)	*0.63 (0.51; 0.78) ****	*0.65 (0.53; 0.79)* *****	*0.69 (0.57; 0.82)****	*0.21 (0.46; 0.08)****
Family affluence (ref. low/medium)	*0.90 (0.67; 1.19)*	*0.91 (0.70; 1.19)*	*0.93 (0.73; 1.18)*	*0.09 (0.26; 0.10)***
BMI	*1.01 (0.98; 1.05)*	*1.01 (0.98; 1.04)*	*1.02 (0.99; 1.05)*	*−0.04 (−0.01; 0.01)*
**Full model results (direct effects)**	*BIC = 3766.55* *CAIC = 3775.55*	*BIC = 3718.42* *CAIC = 3727.42*	*BIC = 4466.80* *CAIC = 4475.80*	*F(7, 716) = 7.84; p < .001, adj. R*^*2*^ *= 0.06*
	**Exp(B), (95% CI)**			**β (B; SE)**
Age	*0.99 (0.92; 1.07)*	*0.98 (0.91; 1.04)*	*0.98 (0.92; 1.04)*	*0.05 (0.03; 0.03)*
Gender	*0.63 (0.51; 0.79) ****	*0.63 (0.52; 0.77) ****	*0.66 (0.55; 0.79)****	*0.22 (0.48; 0.08)****
Family Affluence	*0.92 (0.68; 1.22)*	*0.93 (0.71; 1.23)*	*0.94 (0.74; 1.20)*	*0.10 (0.28; 0.09)***
BMI	*1.00 (0.97; 1.04)*	*1.01 (0.98; 1.04)*	*1.01 (0.98; 1.04)*	*−0.03 (−0.01; 0.01)*
Alcohol consumption	*1.02 (0.98; 1.06)*	*1.02 (0.99; 1.06)*	*1.03 (1.00; 1.06)*	*−0.05 (− 0.01; 0.01)*
Smoking	*1.11 (0.98; 1.25)°*	*1.16 (1.03; 1.30)**	*1.12 (1.01; 1.25)**	*−0.02 (− 0.02; 0.04)*
Perceived sleep duration	*0.87 (0.80; 0.94) ****	*0.87 (0.81; 0.94) ****	*0.91 (0.85; 0.98)***	*0.12 (0.01; 0.03)***
**Full model results (Interaction effects)**	*BIC = 3783.63* *CAIC = 3795.63*	*BIC = 3735.21* *CAIC = 3747.21*	*BIC = 4484.57* *CAIC = 4496.57*	*F(10, 713) = 5.83, p < .001, adj. R*^*2*^ *= 0.06*
	**Exp(B), (95% CI)**			**β (B; SE)**
Age	*0.99 (0.92; 1.06)*	*0.98 (0.91;1.04)*	*0.98 (0.92; 1.04)*	*0.05 (0.03; 0.03)*
Gender (ref. girls)	*0.63 (0.51; 0.79) ****	*0.63 (0.52; 0.78) ****	*0.67 (0.56; 0.80)****	*0.22 (0.47; 0.08)****
Family Affluence (ref. low/medium)	*0.89 (0.65; 1.20)*	*0.90 (0.68; 1.20)*	*0.93 (0.73; 1.20)*	*0.09 (0.26; 0.11)**
BMI	*1.00 (0.97; 1.04)*	*1.01 (0.98; 1.04)*	*1.01 (0.98; 1.04)*	*−0.03 (−0.01; 0.01)*
Alcohol consumption	*1.12 (0.98; 1.25)°*	*1.09 (0.99; 1.21)°*	*1.07 (0.98; 1.16)*	*−0.13 (− 0.01; 0.04)*
Smoking	*1.02 (0.77; 1.37)*	*1.09 (0.82; 1.44)*	*1.15 (0.90; 1.45)*	*−0.11 (− 0.12; 0.10)*
Perceived sleep duration	*0.80 (0.64; 0.99)**	*0.82 (0.70; 0.98)**	*0.90 (0.77; 1.05)*	*−0.00 (− 0.00; 0.06)*
Alcohol * FAS	*0.92 (0.81; 1.04)*	*0.92 (0.83; 1.03)*	*0.96 (0.87; 1.05)*	*−0.02 (− 0.01; 0.04)*
Smoking * FAS	*1.08 (0.79; 1.48)*	*1.06 (0.78; 1.44)*	*0.96 (0.74; 1.25)*	*0.10 (0.12; 0.11)*
Sleep * FAS	*1.09 (0.87; 1.38)*	*1.06 (0.87; 1.29)*	*1.01 (0.85; 1.20)*	*0.14 (0.12; 0.07)*
**Parsimonious model results (direct effects)**	*BIC = 4753.20* *CAIC = 4758.20*	*BIC = 4721.28* *CAIC = 4726.28*	*BIC = 5694.01* *CAIC = 5699.01*	*F(3, 938) = 21.26, p < .001, adj. R*^*2*^ *= 0.06*
	**Exp(B), (95% CI)**			**β (B; SE)**
Gender (ref. girls)	*0.68 (0.56; 0.82) ****	*0.66 (0.55; 0.79) ****	*0.72 (0.61; 0.84)****	*0.19 (0.41; 0.07)****
Family Affluence	*Not included*	*Not included*	*Not included*	*0.10 (0.29; 0.09)****
Smoking	*1.15 (1.05; 1.27)***	*1.17 (1.07; 1.28) ****	*1.16 (1.07; 1.26)****	*Not included*
Sleep	*0.86 (0.80; 0.92) ****	*0.87 (0.82; 0.93) ****	*0.91 (0.86; 0.97)***	*0.15 (0.12; 0.02)****

° *p* ≤ .1; * *p* ≤ 0.05; ** *p* ≤ .01; *** *p* ≤ .001

^a^Gamma generalized linear model

^b^General linear model

**Table 5:** Regression analysis on the moderating role of family affluence in the relation between energy-balance related behaviors and mental health outcomes

	**Dependent variable: mental health outcomes**			
	**Depression**^**a**^	**Anxiety**^**a**^	**Stress**^**a**^	**Self-esteem**^**b**^
**Full model results (direct effects)**	*BIC = 4559.69* *CAIC = 4566.69*	*BIC = 4554.66* *CAIC = 4561.66*	*BIC = 5457.89* *CAIC = 5464.89*	*F(5, 885) = 11.80, p < .001, adj. R*^*2*^ *= 0.06*
	**Exp(B), (95% CI)**			**β (B; SE)**
Age	*1.06 (1.00; 1.12)**	*1.05 (1.00; 1.11)*°	*1.05 (1.00; 1.10)*°	*0.01 (0.00; 0.02)*
Gender	*0.71 (0.58; 0.86)****	*0.69 (0.57; 0.83)****	*0.73 (0.62; 0.86)****	*0.18 (0.39; 0.07)****
Family Affluence	*0.98 (0.75; 1.26)*	*1.01 (0.80; 1.29)*	*0.98 (0.78; 1.21)*	*0.07 (0.20; 0.09)**
Days of breakfast	*0.95 (0.91; 0.99)**	*0.93 (0.89; 0.96)****	*0.94 (0.91; 0.98)****	*0.14 (0.07; 0.02)****
Physical activity days	*0.92 (0.88; 0.97)****	*0.96 (0.92; 1.01)*°	*1.00 (0.96; 1.04)*	*0.04 (0.02; 0.02)*
**Full model results (interaction effects)**	*BIC = 4572.84* *CAIC = 4581.84*	*BIC = 4566.69* *CAIC = 4575.69*	*BIC = 5469.15* *CAIC = 5478.15*	F (7, 883) = 8.70, *p* < .001, adj. *R*^*2*^ = 0.06
	**Exp(B), (95% CI)**			**β (B; SE)**
Age	*1.06 (1.00; 1.12)**	*1.05 (1.00; 1.12)*°	*1.05 (1.00; 1.10)°*	*0.01 (0.00; 0.02)*
Gender (ref. girls)	*0.70 (0.58; 0.86)****	*0.69 (0.58; 0.83)****	*0.73 (0.62; 0.86)****	*0.18 (0.39; 0.07)****
Family Affluence	*0.96 (0.74; 1.25)*	*1.05 (0.82; 1.35)*	*1.01 (0.81; 1.26)*	*0.07 (0.20; 0.10)**
Days of breakfast	*0.97 (0.86; 1.09)*	*0.93 (0.85; 1.03)*	*0.96 (0.88; 1.05)*	*0.06 (0.03; 0.04)*
Physical activity days	*0.93 (0.82; 1.06)*	*0.89 (0.79; 1.01)*°	*0.92 (0.82; 1.03)*	*0.11 (0.06; 0.04)*
Days of breakfast * FAS	*0.96 (0.86; 1.09)*	*0.99 (0.89; 1.11)*	*0.98 (0.89; 1.08)*	*0.09 (0.05; 0.04)*
Physical activity days * FAS	*0.99 (0.86; 1.13)*	*1.09 (0.96; 1.25)*	*1.10 (0.98; 1.24)*	*−0.08 (−0.05; 0.05)*
**Parsimonious model results (direct effects)**	*BIC = 4619.84* *CAIC = 4625.84*	*BIC = 4605.30* *CAIC = 4611.30*	*BIC = 5574.12* *CAIC = 5579.12*	*F (3, 951) = 20.12, p < .001, adj. R*^*2*^ *= 0.06*
	**Exp(B), (95% CI)**			**β (B; SE)**
Age	*1.05 (1.00; 1.11)*°	*1.04 (0.99; 1.10)*	*1.04 (1.00; 1.09)°*	*Not included*
Gender (ref. girls)	*0.69 (0.57; 0.85)****	*0.68 (0.56; 0.81)****	*0.72 (0.61; 0.85)****	*0.18 (0.39; 0.07)****
Family Affluence	*Not included*	*Not included*	*Not included*	*0.08 (0.22; 0.09)**
Physical activity	*0.92 (0.88; 0.97)****	*0.96 (0.92; 1.01)*°	*Not included*	*Not included*
Days of breakfast	*0.95 (0.91; 0.99)**	*0.93 (0.89; 0.97)****	*0.94 (0.91; 0.98)***	*0.15 (0.07; 0.02)****

° *p* ≤ .1; * *p* ≤ 0.05; ** *p* ≤ .01; *** *p* ≤ .001

^a^Gamma generalized linear model

^b^General linear

## Abbreviations

SES: Socioeconomic status
FAS: Family affluence scale
